# Transcriptome-Wide Identification and Characterization of MYB Transcription Factor Genes in the Laticifer Cells of *Hevea brasiliensis*

**DOI:** 10.3389/fpls.2017.01974

**Published:** 2017-11-15

**Authors:** Ying Wang, Di-Feng Zhan, Hui-Liang Li, Dong Guo, Jia-Hong Zhu, Shi-Qing Peng

**Affiliations:** ^1^Key Laboratory of Biology and Genetic Resources of Tropical Crops, Ministry of Agriculture, Institute of Tropical Bioscience and Biotechnology, Chinese Academy of Tropical Agricultural Sciences, Haikou, China; ^2^College of Agriculture, Hainan University, Haikou, China

**Keywords:** *He*v*ea brasiliensis*, MYB-type transcription factor, laticifer, natural rubber, biosynthesis

## Abstract

MYB transcription factors hold vital roles in the regulation of plant secondary metabolic pathways. Laticifers in rubber trees (*He*v*ea brasiliensis*) are of primary importance in natural rubber production because natural rubber is formed and stored within these structures. To understand the role of MYB transcription factors in the specialized cells, we identified 44 MYB genes (named *HblMYB1* to *HblMYB44*) by using our previously obtained transcriptome database of rubber tree laticifer cells and the public rubber tree genome database. Expression profiles showed that five MYB genes were highly expressed in the laticifers. *HblMYB19* and *HblMYB44* were selected for further study. HblMYB19 and HblMYB44 bound the promoters of *HbFDPS1*, *HbSRPP*, and *HRT1* in yeast. Furthermore, the transient overexpression of HblMYB19 and HblMYB44 in tobacco plants significantly increased the activity of the promoters of *HbFDPS1*, *HbSRPP*, and *HRT1*. Basing on this information, we proposed that HblMYB19 and HblMYB44 are the regulators of *HbFDPS1*, *HbSRPP*, and *HRT1*, which are involved in the biosynthesis pathway of natural rubber.

## Introduction

MYB transcription factors (TFs) comprise a TF family highly rich in plants (Dubos et al., 2010). Plant MYB TFs contain highly conserved MYB domains involved in DNA binding (Rosinski and Atchley, 1998; Jin and Martin, 1999). On the basis of the number of MYB repeats present in their sequences, MYB TFs are divided into the following four groups: 1R-MYB, 2R-MYB, 3R-MYB, and 4R-MYB (Rosinski and Atchley, 1998; Jiang et al., 2004). Each MYB repeat contains approximately 52 amino-acid residues, which form three α-helices (Kanei-Ishii et al., 1990). Since the first plant MYB gene *ZmC1* was characterized from *Zea mays* (Paz-Ares et al., 1987), numerous MYB genes have been identified and characterized from plants (Stracke et al., 2001; Dubos et al., 2010; Katiyar et al., 2012; Hou et al., 2014; Wang et al., 2015; Zhou et al., 2015; He et al., 2016). At least 155 and 197 MYB genes have been identified in rice and *Arabidopsis*, respectively (Katiyar et al., 2012). MYB TFs are involved in plant growth and development (Cominelli and Tonelli, 2009; Oh et al., 2011; Huang et al., 2013; Cai et al., 2015), hormone signal transduction (Shin et al., 2007; Zhao et al., 2014), secondary metabolism (Chezem and Clay, 2016; Chezem et al., 2016; Zhai et al., 2016), abiotic stress responses (Dai et al., 2007; Oh et al., 2011; Peng et al., 2011), and disease resistance (Liu et al., 2013; Zhang et al., 2015).

In rubber tree (*Hevea brasiliensis*), natural rubber (NR) is obtained from latex, which constitutes the cytoplasmic content of laticifer cells (Kush, 1994). Laticifers are specialized cells located inside the phloem tissue of rubber trees (Kush, 1994; Hao and Wu, 2000). NR is synthesized in the rubber particles of laticifers (Kush et al., 1990). Laticifers are of primary importance in NR production. However, the biological functions of the rubber molecule and latex remain unclear (Ko et al., 2003). The regulatory mechanisms of NR biosynthesis are also poorly understood (Li et al., 2016b; Tang et al., 2016; Yamashita et al., 2016). MYB TFs play vital roles in regulating plant secondary metabolic pathways, such as the general phenylpropanoid pathway and lignin, flavonoid, and glucosinolate pathways (Chezem et al., 2016). However, few MYB TF genes related to the NR biosynthesis pathway in rubber trees have been reported. To understand the MYB TFs in laticifers, we identified and characterized 44 *MYB* genes (named *HblMYB1* to *HblMYB44*) in this study. We found that five MYB genes were more highly expressed in laticifers than in other tissues. Furthermore, HblMYB19 and HblMYB44 may be the regulators participating in the NR biosynthesis pathway.

## Materials and Methods

### Plant Materials

Two-year-old trees of *H. brasiliensis* clones of Wenchang11 were grown in the Rubber Research Institute of Hainan Agricultural Reclamation, Wenchang, Hainan, China. Rubber tree shoots were treated by 0.07% methyl jasmonate (JA), 0.5% Ethrel (ET), 200 μm abscisic acid (ABA), and 200 μm salicylic acid (SA) as described previously (Hao and Wu, 2000). Then, seven groups of 10 trees were used in each treatment, in which the plant growth regulator was applied at 1, 3, 6, 9, 12, 24, and 48 h before tapping. One group served as an untreated control. After the treatments at all time points, latex samples from all the tested trees were collected and mixed together thoroughly. The resulting solution was then divided into five equal volumes for RNA extraction (Tang et al., 2007). The other tissues (leaves, barks, roots, and flowers) of the rubber tree were then collected and stored in liquid nitrogen for RNA extraction.

### DNA and RNA Extraction

DNA was extracted from young leaves of *H. brasiliensis* through the cetyl trimethylammonium bromide method (Allen et al., 2006). The total RNA from latex was isolated in accordance with Tang’s method (Tang et al., 2007), whereas that from the other tissues was extracted as described previously (Li et al., 2011). Three biological replicates were used for RNA extraction.

### Identification of *MYB* in the Laticifer Cells

The analytical software NCBI-Blast-2.2.28+-win32 and the genomic data of *H. brasiliensis* (Rahman et al., 2013; Tang et al., 2016) were downloaded from the National Center for Biotechnology Information (NCBI^1^). They were used to establish a local *H. brasiliensis* genome database. The *MYB* unigenes were obtained from the transcriptome database of the rubber tree latex in our previous study (Li et al., 2016a). The *MYB* unigenes were employed as query sequences for a BLAST search in the local rubber tree genome database. All candidate MYB genes were further analyzed for confirmation by using the NCBI Conserved Domain Search database^2^. The physical and chemical properties of HblMYB were analyzed with ExPASy^3^), and the CDS of *HblMYBs* were analyzed with GSDS^4^. The predicted amino-acid sequences of HblMYBs were aligned with ClustalX. The highly conservative domains of HblMYB proteins were illustrated with espript^5^, and the 3D structure was constructed with SWISS-MODEL^6^.

### Phylogenetic Tree Analysis

*Arabidopsis* MYB protein sequences were downloaded from the phytozome^7^. *Arabidopsis* MYB protein sequences and the deduced amino-acid sequences of HblMYBs were aligned using ClustalX. Then, using the neighbor-joining method and the MEGA6.0 program, we constructed the phylogenetic tree between HlMYBs and the known MYB from *Arabidopsis*, and bootstrap analysis was conducted with 1,000 replicates (Yang et al., 2015).

### Quantitative Real-Time PCR (qRT-PCR)

The cDNA synthesis for qRT-PCR was performed with a RevertAid^TM^ First-Strand cDNA Synthesis Kit (Fermentas, Lithuania). qRT-PCR was performed using a SYBR Premix EX Taq Kit (TaKaRa, Japan). The primers for the *HblMYBs* were designed using the Primer Premier 5 software (Supplementary Table S1). *HbACT7* was amplified through the following primers as standard control: 5′-TGTCAGCAACT GGGACGATATGG-3’ as primer 1 and 5′-GAGTCATCTTCTCTCTGTTGGC-3′ as primer 2 (Li et al., 2016a). qRT-PCR was performed as follows: 3 min at 95°C for denaturation, 40 cycles for 10 s at 95°C, 20 s at 58°C, and 25 s at 72°C. The quantitative value obtained from qRT-PCR is considered the cycle threshold (Ct). The normalized expression values for each gene were calculated through the following formula: 2^-(Ct[gene]-Ct[HbACT7])^, in which *HbACT7* was used as a housekeeping gene for normalization. Three individual reactions were replicated. Data were analyzed by ANOVA to analyze the significant differences on the basis of Fischer’s LSD test (*P* < 0.05 and *P* < 0.01; Quirk et al., 2016).

### Subcellular Localization

The open reading frames (ORFs) of *HblMYBs* were amplified by PCR using primers (Supplementary Table S1). PCR products were ligated into the pCAMBIA1302 vector, generating pHblMYBs-GFP. pCAMBIA1302 and pHblMYBs-GFP were introduced into the onion epidermis by *Agrobacterium*-mediated transformation. The transformed onion epidermis was then cultured on an MS solid medium in the dark at 26°C for 5 h and then observed with a confocal microscope (Zeiss LSM510, Germany).

### Yeast One-Hybrid Assay

The *HbFDPS1* promoter (1,066 bp) and *HbSRPP* promoter (1,735 bp) were amplified by PCR with the primers as described previously (Guo et al., 2010, 2014). The 1,136 bp *HRT1* promoter was amplified using the primers (Supplementary Table S1) based on the *HRT1* sequence from the rubber tree genome database (GenBank accession: LVXX01000000; Tang et al., 2016). These promoters were cloned into the *Spe*I/*Mlu*I sites of the pHiS2.1 vectors (Clontech) to form the bait vectors pHiS-pHbSRPP, pHiS-HbFDPS1, and pHiS-HRT1. The ORFs of *HbMYB19* and *HbMYB44* were fused into the GAL4 domains of the pGAD7 vectors to generate the prey constructs pGAD-HbMYBs. The bait and prey vectors were then transformed into the yeast strain Y187 (Clontech). Afterward, the introduced yeast was cultivated on an SD/-Trp/-His/-Leu medium supplemented with 70 mM 3-amino-1,24-triazole (3-AT) at 30°C for 3 days.

### Dual-Luciferase (Dual-LUC) Assay

The Dual-LUC assay was performed as described previously (Hellens et al., 2005). In brief, the promoters of *HbSRPP, HbFDPS1*, and *HRT1* were cloned into pGreenII 0800 vectors, in which the expression of *Renilla* luciferase (REN-Luc) provided an internal control (Hellens et al., 2005). The ORFs of *HbMYB19* and *HbMYB44* were amplified with the primers (Supplementary Table S1) and then with the inserted pGreenII 62Sk vectors. All constructs were introduced into the *Agrobacterium tumefaciens* strain GV3103. The introduced GV3103 harboring pGreen-pHbSRPP, pGreen-pHbFDPS1, or pGreen-pHRT1 were mixed with the introduced GV3103 harboring pGreenII 62Sk-HblMYBs in a volume ratio of 1:5. The mixtures were injected into tobacco leaves. After culturing for 3 days, the infected areas of the leaves were obtained by puncher, and the protein was extracted. The activities of the luciferase and REN-Luc were measured through the Dual-LUC Reporter Assay System in accordance with the manufacturer’s manual (Promega). The binding ability of the HblMYBs to the promoters of *HbSRPP*, *HbFDPS1*, and *HRT1* were represented by LUC/REN (ratio of LUC to REN-Luc). Three biological repeats were measured. The data were analyzed by ANOVA to determine the significant differences on the basis of the Fischer’s LSD test (*P* < 0.05 and *P* < 0.01) (Quirk et al., 2016).

## Results

### Identification and Sequence Conservation Analysis of HblMYBs

**FIGURE 1:**
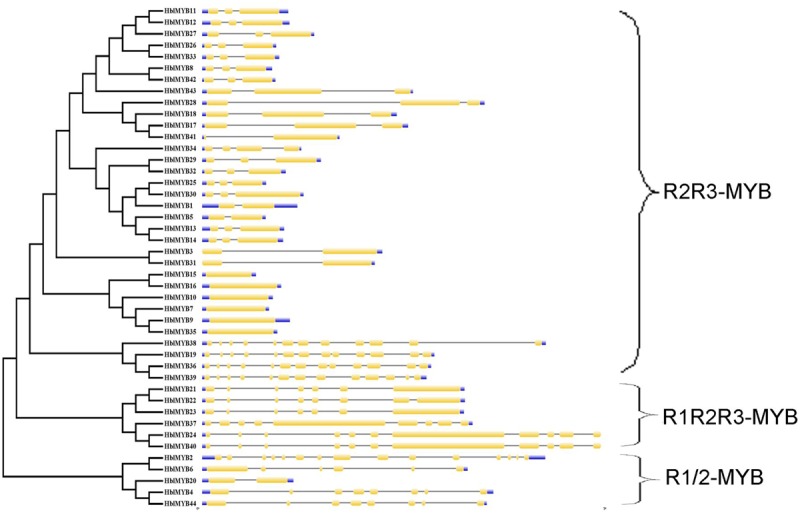
Exon–intron structure of *HblMYBs* based on their evolutionary relationships. The NJ evolutionary tree was generated with 1,000 bootstrap replicates based on the full-length sequences of the HblMYBs (left side). Exon–intron analyses of the *HblMYBs* were performed with GSDS. Meanwhile, the intron–exon structures of *HblMYBs* are described in the right portion. The exons and introns are indicated by yellow boxes and single lines, and the 5′-UTR or 3′-UTR are indicated by blue boxes. The lengths of the exons and introns for the corresponding *HblMYBs* are shown proportionally.

The transcriptome database of the rubber tree latex was obtained in our previous study (Li et al., 2016a). A total of 76 *MYB* unigenes were obtained using our previously established latex transcriptome database. A total of 44 *MYB* genes were confirmed in the present study after the 76 *MYB* unigenes were searched in BLAST against those from the public rubber tree genome database. These MYB genes were named as *HblMYB1* to *HblMYB44.* The sequences and properties of the identified 44 *HblMYBs* from the laticifer cells of the rubber tree are listed in Supplementary Table S2. The genomic structure of the 44 *HblMYB* genes was analyzed by GSDS. The 44 *HblMYBs* vary with respect to exon–intron gene structure. In particular, six *HblMYBs* contain only one exon, six *HblMYBs* contain two exons and one intron, 17 *HblMYBs* contain three exons and two introns, and the other *HblMYBs* containing more than five exons (**Figure 1**).

**FIGURE 2:**
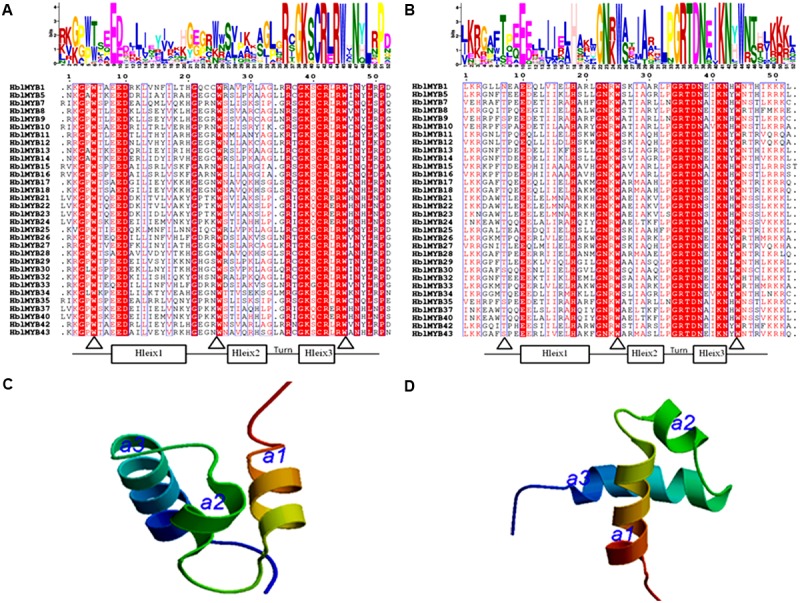
Predicted domains in the HblMYBs. The conserved domains were determined by MEME through the protein sequences of the HblMYBs. This online software was used to create the logo representations of the R2 **(A)** and R3 domains **(B)**. The *y*-axis (measured in bits) depicts the overall height of the stack and indicates the sequence conservation at that position. Meanwhile, the symbol height within the stack indicates the relative frequency of each amino acid at that position. The predicted 3D structure models of the R2 **(C)** and the R3 domains are presented **(D)**. The R2 and R3 domains of HblMYB1 were utilized to construct structural models.

The number of amino acids in the HblMYB proteins ranges from 246 (HblMYB26, HblMYB33) to 1,043 (HblMYB24); protein molecular weights, from 27.24 (HblMYB10) to 115.12 (HblMYB24); and pI, from 5 (HblMYB37) to 9.44 (HblMYB3; Supplementary Table S2). Further analysis showed that the MYB domain (R unit) was highly conserved in the N-terminus. Among all the 44 HblMYBs, 5 HblMYBs contained one R unit, 33 HblMYBs had two R units, and 6 HblMYB contained three R units (**Figure 1**). The R2 repeats of R2R3-HblMYBs harbored three highly conserved tryptophan residues (W) at positions 6, 26, and 46 (**Figure 2A**). By contrast, the tryptophan residues were highly conserved at positions 25 and 44 of the R3 repeats (**Figure 2B**). Other highly conserved residues, except the tryptophan residues, were confirmed in the R2 and R3 domains. These residues included Gly (G4), Glu (E10), Asp (D11), Gly (G22), Arg (R37), Gly (G39), Lys (K40), Cys (C42), Arg (R43), Arg (R45), Asn (N48), Leu (L50), and Pro (P52) in the R2 repeat (**Figure 2A**), as well as Glu (E10), His (H18), Gly (G22), Asn (N23), Gly (G34), Arg (R35), Thr (T36), Asp (D37), Asn (N38), Lys (K41), and Asn (N42) in the R3 repeat (**Figure 2B**). Additionally, the 3D protein structure prediction showed that the R2 and R3 domains of the HblMYB proteins formed three α-helices (**Figures 2C,D**), which participate in transcriptional regulation (Dubos et al., 2010).

### Phylogenetic Analysis of the HblMYBs

**FIGURE 3:**
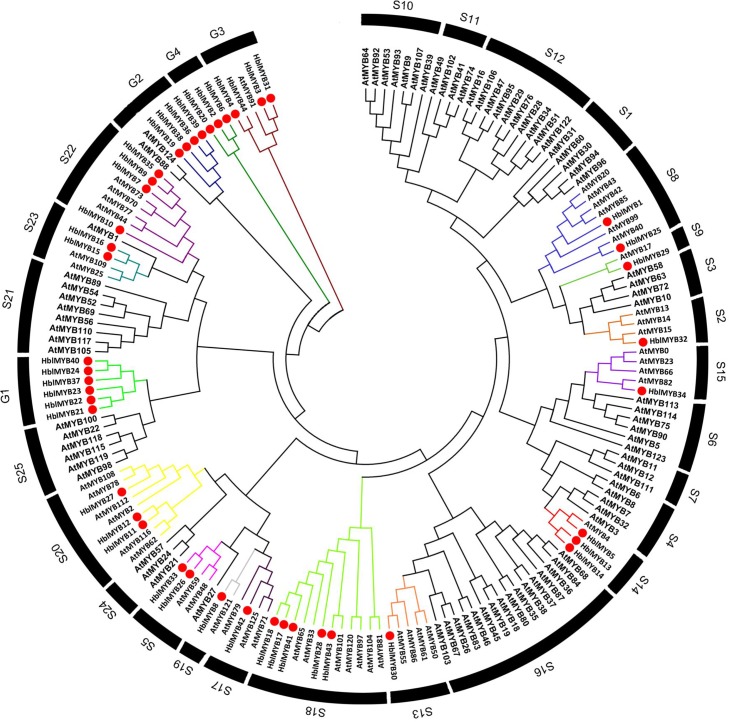
Phylogenetic analysis of HblMYBs with *Arabidopsis* MYB TFs by MEGA6.0 from CLUSTALW alignments. The *Arabidopsis* MYB TFs used in the evolutionary analysis were retrieved from the phytozome (https://phytozome.jgi.doe.gov/pz/portal.html), and the internal branch support was estimated with 1,000 bootstrap replicates.

To infer the evolutionary relationships between HlMYBs and the known MYB from *Arabidopsis*, we constructed a phylogenetic tree between the obtained 44 HblMYBs and 126 *Arabidopsis* MYB TFs (**Figure 3**). All the 170 MYB were classified into 29 subgroups. Meanwhile, 44 HblMYB proteins were divided into 17 subgroups (S2, 5, 8, 9, 13, 14, 15, 17, 18, 19, 20, 22, and 23 and G1, 2, 3, and 4). Their orthologous MYBs were from *Arabidopsis*. This result suggests the existence of few closely related orthologous MYBs between rubber trees and *Arabidopsis*. Of the 29 subgroups, 10 subgroups (S1, 4, 6, 7, 10, 11 12, 16, 21, and 25) did not exhibit any rubber tree ortholog, and two subgroups (G1 and G4) did not present any *Arabidopsis* ortholog. The phylogenetic tree indicated the existence of an ancestral set of MYB genes prior to the divergence of rubber tree and *Arabidopsis*.

### Expression of *HblMYBs* in Different Tissues

**FIGURE 4:**
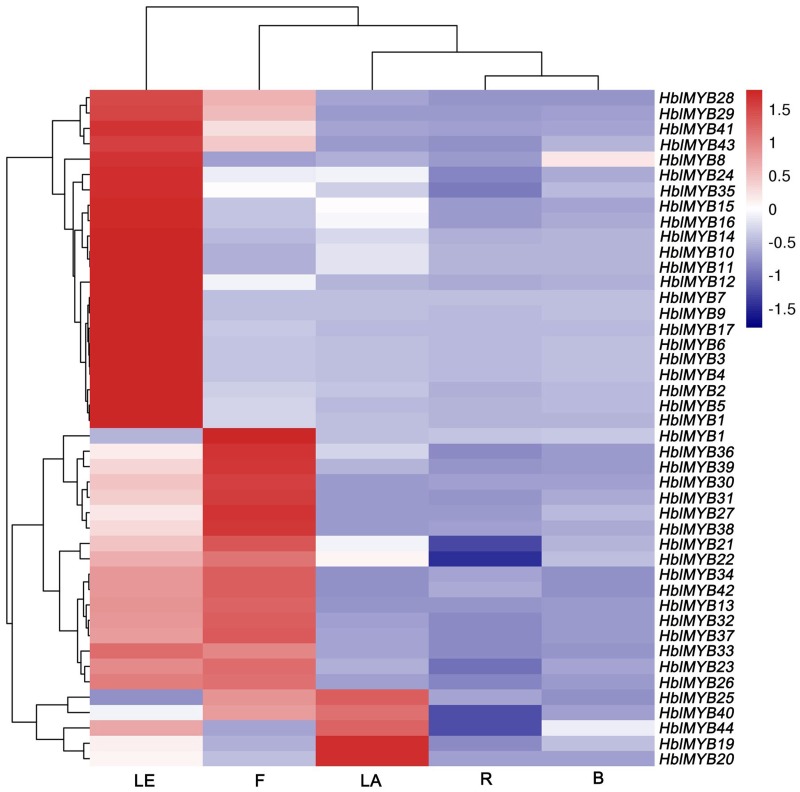
Expression patterns of *HbMYBs* in different tissues. The heatmap was created using log_2_-based values from three replicates of qRT-PCR data. The scale represents the relative signal intensity values. R, root; B, bark; LE, leaf; F, flower; LA, latex.

The expression patterns of 44 *HblMYBs* were detected in roots, barks, leaves, flowers, and latex by qRT-PCR (**Figure 4**). The results showed that the expression profiles of the 44 *HblMYBs* differed across different tissues. Five *HblMYBs* (*HblMYB19*, *20*, *25*, *40*, and *44*) showed higher transcription levels in latex, whereas 36 *HblMYBs* in the leaves, and 22 *HblMYBs* in the flowers. By contrast, all *HblMYBs* clearly presented with lower expression in the barks and roots.

### Expression Patterns of *HblMYBs* in the Latex Respond to Phytohormone

**FIGURE 5:**
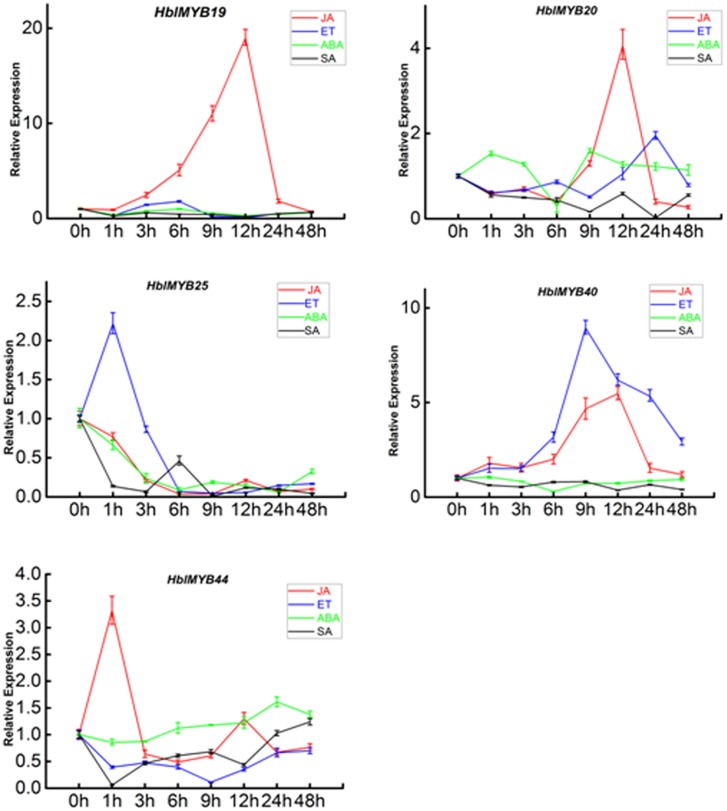
Expression patterns of the five *HbMYBs* responding to phytohormone treatment. The relative transcript abundances of *HbMYBs* were examined via qRT-PCR. The *y*-axis is the scale of the relative transcript abundance level. The *x*-axis shows the time course of the phytohormone treatment. The average of three independent biological replicates was computed at each time. Data are presented as mean ± standard error (SE) (*n* = 3). The statistical significance of the differences was assessed by ANOVA (one or two stars correspond to *P* < 0.05 and *P* < 0.01, respectively).

Given the expression of *HblMYBs* in different tissues, five *HblMYBs* (*HblMYB19*, *20*, *25*, *40*, and *44*, which showed high expression levels in latex), were selected for further analysis on their response to exogenous phytohormone. Overall, the five *HblMYBs* showed different expression patterns under MeJA, ET, ABA, and SA treatments (**Figure 5**). Results showed that JA induced the expression of *HblMYB19*, *20*, *40*, and *44* but down-regulated that of *HblMYB25* at the 6 h time point. ET treatment upregulated the transcript abundance of *HblMYB20*, 25, and *40* but downregulated those of *HblMYB19* and 44 at the 12 or 9 h time point. ABA stress induced the expression of *HblMYB44* at 24 h time point. However, we repressed the *HblMYB19*, *20*, *25*, and *40* expression at 12 or 6 h time point. Lastly, SA treatment repressed the *HblMYB20, 25*, and *44* expression at 24, 9, or 3 h time point, but did not significantly affect the *HblMYB19* and *44* expression.

### Subcellular Localization of HblMYB19 and HblMYB 44

**FIGURE 6:**
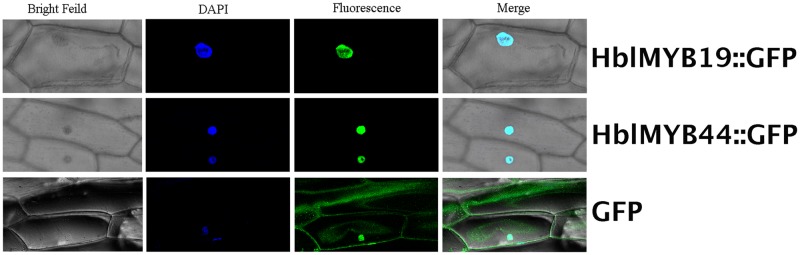
Nuclear localization of HbMYB19 and HbMYB44. The upper panel shows the corresponding bright-field image, DAPI image, fluorescence image, and merged image of HbMYB19-GFP. The middle panel displays the corresponding bright-field image, DAPI image, fluorescence image, and merged image of HbMYB44-GFP. The lower panel shows the corresponding bright-field image, DAPI image, fluorescence image, and merged image of GFP.

Given the expression of *HblMYBs* and response to JA in laticifers, *HblMYB19* and *HblMYB44* were selected for further analysis. First, subcellular localization analysis was performed on HblMYB19 and HblMYB44. We found that the GFP signals expressed the fusion proteins of HblMYB19, and 44 were present only in the nucleus of onion epidermal cells. By contrast, the GFP signals expressing the GFP protein were present obviously both in the nuclei and cytosol (**Figure 6**).

### Activation of the Promoter of *HbFDPS1, HbSRPP*, and *HRT1* by HblMyb19 and HblMyb44 in Yeast

**FIGURE 7:**
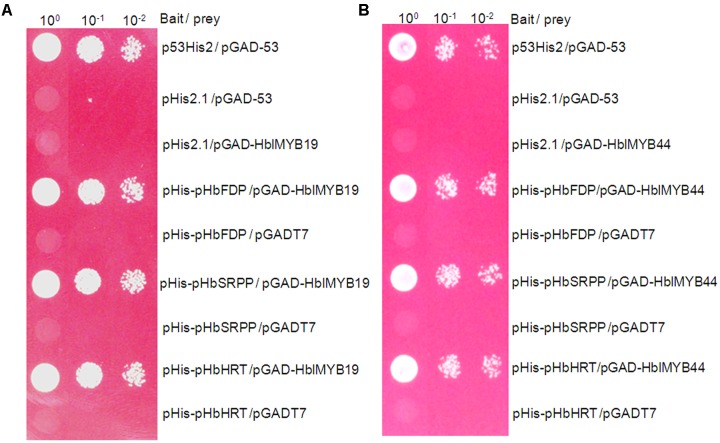
Activation of the promoters of *HbFDPS1*, *HbSRPP*, and *HRT1* by HbMYB19 **(A)** and HbMYB44 **(B)** in yeast. Yeast cells carrying the bait vector and prey vector were grown in SD/-Leu selective medium containing 470 mM AbA at 30°C for 3 days.

Farnesyl diphosphate synthase (FDPS), small rubber particle protein (SRPP), and *Hevea* cis-prenyltransferases or rubber transferase (HRT) from *H. brasiliensis* are responsible for the *cis*-1,4-polymerization of isoprene units from isopentenyl diphosphate (IPP) and implicated in NR yield (Light et al., 1989; Oh et al., 1999; Asawatreratanakul et al., 2003). To determine whether HblMybs bind the promoters of *HbFDPS1*, *HbSRPP*, and *HRT1*, we performed a yeast one-hybrid analysis. The yeast clones harboring pHblMyb19+pHIS2-pHbSRPP, pHblMyb19+pHIS2-p*HRT1*, pHblMyb19+pHIS2-*pHbFDPS1*, pHblMyb44+pHIS2-HbSRPP, pHblMyb44+pHIS2-p*HRT1*, and pHblMyb44+pHIS2-*pHbFDPS1* can grow on SD/-Trp/-His/-Leu selective medium added with 70 mM 3-AT (**Figure 7**). This result indicated that HblMyb19 and HblMyb44 bound the promoters of *HbSRPP*, *HRT1*, and *HbFDPS1* in yeast.

### Activation of the Promoters of *HbFDPS1, HbSRPP*, and *HRT1* by HblMyb19 and HblMyb44 in Plants

**FIGURE 8:**
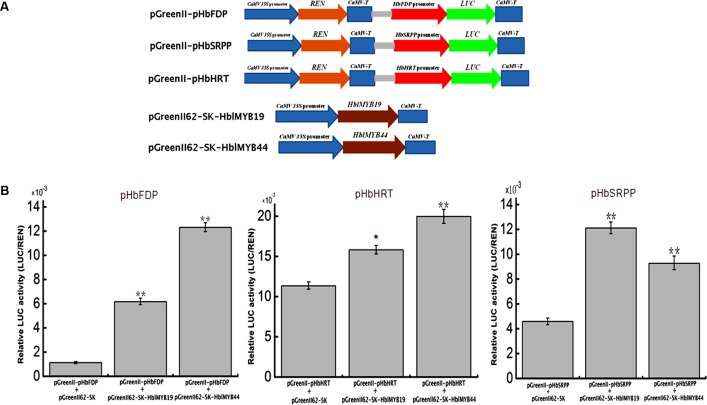
Activation of the promoters of *HbFDPS1*, *HbSRPP*, and *HRT1* by HblMyb19 and HblMyb44 in a transient expression system. **(A)** Schematics of the transient expression vectors used in the transient expression analysis. **(B)** Relative LUC activity from the transient expression analysis of the promoters of *HbSRPP*, *HRT1*, and *HbFDPS1* co-infiltrated with a plasmid containing the genes for HblMyb19 and HblMyb44 fused with the 35S promoter. Three replicates were included for each sample. Data are presented as mean ± SE (*n* = 3), and the statistical significance of the differences was assessed by ANOVA (one or two stars correspond to *P* < 0.05 and *P* < 0.01, respectively).

Given the interaction between yeast HblMyb19 and HblMyb44 and the promoters of *HRT1, HbSRPP*, and *HbFDPS1*, we investigated whether HblMyb19 and HblMyb44 participate in the regulation of the promoters of *HbHRT, HbSRPP*, and *HbFDPS1* in plants. For this purpose, HblMyb19 and HblMyb44 were transiently expressed in tobacco by *Agrobacterium*-mediated transformation (**Figure 8A**). The luciferase activity controlled by the HblMyb19 or HblMyb44 binding of the promoters of *HRT1, HbSRPP*, and *HbFDPS1* was elevated (**Figure 8B**). Moreover, the expression of HblMyb19 or HblMyb44 resulted in a significant increase in luciferase activity. The data showed that the transient expression of HblMyb19 and HblMyb44 activated the promoters of *HRT1*, *HbSRPP*, and *HbFDPS1*.

## Discussion

Plant MYBs regulate secondary metabolism (Liu et al., 2015; Chezem et al., 2016). Several flavonoid-related MYB TFs were already identified in plants. TT2, the first identified proanthocyanidin (PA)-related MYB TF, induces the biosynthesis of PAs in seed coats of *A. thaliana* by activating DFR, ANS, and ANR (Nesi et al., 2001). In grapevines, VvMYBA1 and VvMYBA2 are specific regulators, which activate the *UFGT* of the anthocyanin pathway (Kobayashi et al., 2002). MdMYB10 alleles are the key regulatory factors during the coloration of apple fruits (Takos et al., 2006; Espley et al., 2007). *VvMYBPA2* was identified as a direct regulator of several structural flavonoid pathway genes in grapevines (Terrier et al., 2009). MdMYB3 activates some flavonoid-biosynthesis-related genes, such as *CHI, CHS, FLS*, and *UFGT*, in apple fruits (Vimolmangkang et al., 2013). In strawberry, FaMYB10 regulates the anthocyanin-pathway-related genes, including most of the EBGs and LBGs in ripened fruit receptacles during ripening (Medina-Puche et al., 2014). Few MYB genes from *H. brasiliensis* have been reported. Overexpressed *HbMyb1* in tobacco suppresses stress-induced cell death (Peng et al., 2011). Another MYB gene down-regulated in trees with tapping panel dryness was identified from the SSH library (Venkatachalam et al., 2007). The MYB gene was significantly induced by ET, ABA, JA, SA, and wounding treatments (Qin et al., 2014). To date, whether MYB TFs help regulate the NR synthesis pathway in rubber trees remains unknown. NR is synthesized from the precursor IPP (Archer and Audley, 1987; Madhavan et al., 1989; Cornish and Backhaus, 1990; Chow et al., 2007, 2012; Sando et al., 2008). During NR biosynthesis, FDPS and HRT, along with SRPP, are critical to NR biosynthesis and frequently used to determine the efficiency of a process (Light et al., 1989; Oh et al., 1999; Asawatreratanakul et al., 2003). However, the regulatory mechanism of rubber biosynthesis is incompletely understood (Yamashita et al., 2016; Tang et al., 2016). HbWRKY1 was identified as a negative transcription regulator of *HbSRPP* (Wang et al., 2013). Other WRKY proteins may participate in the regulation of NR biosynthesis (Li et al., 2014). HbMADS4 was also found as a negative transcriptional regulator of *HbSRPP* (Li et al., 2016b). HbCZF1, a CCCH-type zinc-finger protein, highly activates the *hmg1* promoter, and HbCZF1 may help regulate NR biosynthesis (Guo et al., 2015). In the present study, HblMyb19 and HblMyb44 bind the promoters of *HRT1, HbSRPP*, and *HbFDPS1* in yeast. Moreover, HblMyb19 and HblMyb44 activated the promoters of *HRT1*, *HbSRPP*, and *HbFDPS1* in plants. These results strongly indicated that *HRT1, HbSRPP*, and *HbFDPS1* are the target genes of HblMyb19 and HblMyb44, and HblMyb19 and HblMyb44 are transcriptional activators of *HRT1, HbSRPP*, and *HbFDPS1.* Moreover, JA signaling may regulate NR biosynthesis in laticifers (Zeng et al., 2009; Pirrello et al., 2014). The induction of the expression of *HblMyb19* and *HblMyb44* by MeJA showed that HblMyb19 and HblMyb44 may play a role in the JA signaling pathway. Additionally, HRT1, HbSRPP, and HbFDPS1 can be utilized to increase the NR content in rubber tree. As a result, upregulating *HRT1*, *HbSRPP*, and *HbFDPS1* can improve NR productivity in the transgenic plants. The identification and characterization of the NR-biosynthesis-related MYB TFs would greatly help increase the understanding of the molecular mechanism of NR metabolism.

## Conclusion

In the present study, 44 *MYB* genes (named *HblMYB1* to *HblMYB44*) were identified form rubber tree laticifer cells, and we found that five genes were highly expressed in laticifers. HblMYB19 and HblMYB44 bind the promoter of *HbSRPP*, *HRT1*, and *HbFDPS1* in yeast. Furthermore, the transient over-expression of *HblMYB19* and *HblMYB44* in tobacco plants significantly increased the activity of the promoters of *HbSRPP*, *HRT1*, and *HbFDPS*. Basing on all the above-mentioned information, we propose that HblMYB19 and HblMYB44 are regulators of *HbSRPP*, *HRT1*, and *HbFDPS1*, all of which participate in NR biosynthesis.

## Author Contributions

S-QP and YW designed the research, YW, D-FZ, H-LL, DG, and J-HZ performed the research, and YW and S-QP wrote the paper. All authors read and approved the final manuscript.

## Conflict of Interest Statement

The authors declare that the research was conducted in the absence of any commercial or financial relationships that could be construed as a potential conflict of interest.

## Footnotes

1 http://www.ncbi.nlm.nih.gov/

2 http://www.ncbi.nlm.nih.gov/Structure/cdd/wrpsb.cgi

3 http://web.expasy.org/compute_pi/

4 http://gsds.cbi.pku.edu.cn/

5 http://espript.ibcp.fr/ESPript/cgi-bin/ESPript.cgi

6 https://swissmodel.expasy.org/interactive

7 https://phytozome.jgi.doe.gov/pz/portal.html
