# Supplementary material for: Transcriptome-Wide Identification and Characterization of MYB Transcription Factor Genes in the Laticifer Cells of Hevea brasiliensis
Source: Front Plant Sci. 2017 Nov 15;8:1974. doi: 10.3389/fpls.2017.01974 (PMC5694821; doi:10.3389/fpls.2017.01974)
Supplement: Supplementary file 1 [file Table_1.DOC]

Table S1. Primers used in this study

| Purpose | Gene or vector | Forward primer（5’-3’） Reverse primer（5’-3’） | |
| --- | --- | --- | --- |
| qRT-PCR | *HblMYB1* | GGAGATGACTACTTTGGACTTGG | AAGGCCTGGTTCCTTGTTAG |
|  | *HblMYB2* | CTCCATCATCACCAGCTCATAA | ACATCCTTTGAGGCCCTTATC |
|  | *HblMYB3* | GAGCACAATGGCCGATAAGA | GTCCAAGCCATAAGCAGACA |
|  | *HblMYB4* | CTGTTGGCCCTTACAGCAAT | GGACAGATGCTTCCCAAGAC |
|  | *HblMYB5* | GCAACTCTGGCTATGACTTCT | CCCTTTCTCATTTCTCTCCATTTC |
|  | *HblMYB6* | CAGGGTATCAATTTGCACCTTAC | GCATCTCAATACCTTGACAAACC |
|  | *HblMYB7* | TAGGAATGTGGCCATGAATCG | TCCTCTCTCTCTCTCTCTCTCT |
|  | *HblMYB8* | CAGCAAGATTGCCAAGCAAA | ACCAGTTAGACACAAGCACAA |
|  | *HblMYB9* | GCCATAAGGAACGCTGTAGTTA | CGACTCAGCTGGATCATCTTG |
|  | *HblMYB10* | GAGGGAAGTGCGAGAGTATATG | CAACACCACAGTTGTTTGTTTC |
|  | *HblMYB11* | GAGGTAACATATGGAGCATGGA | GACATGGAATTAACAGTTGCTGTA |
|  | *HblMYB12* | CAGAATCCACTCTGCGATCC | TCCATGCTCCATATGTTACCTC |
|  | *HblMYB13* | ATTGGGTTCACAGAGGAGTG | CATTTGCACCGATCTAACCC |
|  | *HblMYB14* | GGGTTCACAGAGAAGTGAATTG | CAATCTAACCACAACTCCCAAC |
|  | *HblMYB15* | CCTATGGATCTCAAGCGCAAAT | GGTTGCGACACCCATTAACT |
|  | *HblMYB16* | GCACCTCATGGATCTCAAGC | GGTTGCGACACCCATTAACT |
|  | *HblMYB17* | GATGGAGGCTTGGTTCTTGT | GGACCTGACTACCCAATTGAAG |
|  | *HblMYB18* | GCCTGCTGTCTGTCAAATGTC | GCACAGACCTGACTACCCAA |
|  | *HblMYB19* | AGAAGAGCCCTCCTCCAAAG | CTGCAATCCTAGGAAACTGACATT |
|  | *HblMYB20* | GCCTAATTTGGAGTTCACCTTG | CTCTTCATCACCAATCCCTTCT |
|  | *HblMYB21* | GCGCTTGTTTCTGAAGGTTG | ACTGTCAGCCAGTCTTTGAG |
|  | *HblMYB22* | GCGCTTGTTTCTGAAGGTTG | ACCAGTAGGAAACTGTCAGC |
|  | *HblMYB23* | GTGAGAGCACCAATTCTGTGA | GCAGATGAAGGAGAAGGGATTT |
|  | *HblMYB24* | GAATGCCGTACCCTTGACTT | CAACAGATAGGAAGAGGGACTTG |
|  | *HblMYB25* | GCTGTCATGGGATGGGTTTA | ACAGGCAGAAAGATGGACAA |
|  | *HblMYB26* | GGGTTTAGTGAACAAGGTTGC | TCTTACCCTCCTCTTCATCCA |
|  | *HblMYB27* | TCAATCTGGGCAAGTTGGCT | CTGCTCCATGGCTTGGAAATC |
|  | *HblMYB28* | GACAAGGAAGCTCCCAATCAG | CACCAAATGCAGACCCTACTG |
|  | *HblMYB29* | TGCCATTGCCTCTCAACTAC | GAGTCAAGTCCCATGCAAAGA |
|  | *HblMYB30* | GAACTACTGCCGTACAGAACC | GCCAACTAGTACTTGACCCTTC |
|  | *HblMYB31* | GAGAAATCAAGCCGGAGGAG | CGTTTCCGCATCTCTTCTCAA |
|  | *HblMYB32* | CCAAGATCTTTGGTCAGAAGCA | GCTGCTACCATACTGTCTGATG |
|  | *HblMYB33* | TCAATTGCATCTCCCTCATGG | GGAAGAAACATCTTACCCTCCTC |
|  | *HblMYB34* | CCCTCAGGATGAAGCTGTAAG | CTGGCCATGTCAAATGGTAATG |
|  | *HblMYB35* | GAGATGATACGGAAGGAAGTGAG | CGTTCCTTATGGCTTCAGTTTG |
|  | *HblMYB36* | CTGTTTCTGCCTCATTAAGTGC | CCTGCTGCTAAGGATCTGAA |
|  | *HblMYB37* | GTTTCGACGTCAACTGAATGC | CAACAGATAGGAAGAGGGACTTG |
|  | *HblMYB38* | CTGGACTTTCCAGATCATTGAG | CAACTGCTGCTGATGACTTG |
|  | *HblMYB39* | CTGTTTCTGCCTCATTAAGTGC | CCTGCTGCTAAGGATCTGAA |
|  | *HblMYB40* | CAGAATGCCGTACCCTTGAC | CCTGCAACCCTTCAACAGATAG |
|  | *HblMYB41* | CTTGCTGAAAGTTCTGCCTTG | TATAGGAGGGCACTCAGTGAA |
|  | *HblMYB42* | CGAGGAATTTCTCTGGGATGG | ATGCTAGCCTTGCTAGATGC |
|  | *HblMYB43* | ATCCAATGGGCCATCTTCAG | CCGGTAAGTTATCCCAAGAGC |
|  | *HblMYB44* | GTCCCGAGTTTGAGGATCATAG | CTTCTCTTGGAGCCGAAAGG |
|  |  |  |  |
|  | pc1302-HblMYB19 | CCCATGGGTATGCAAGAACCAAAGACGAAGAG | GGACTAGTTTATAGACTTTGGAGGAGGGCTC |
|  | pc1302-HblMYB44 | CCCATGGGTATGGTGTCGGTGAACCCCAACCCG | GGACTAGTTCAGCCTCTCACTTCTCTTGGAGC |
| Vector constrction | pHis-HbSRPP | AGCACTAGTTCATAGTTGTTTATTTCATCCCATA | GCCTGCGCATAAAACTAAAACAAATCTCTCTATT |
|  | pHis-HbFDP | CACTAGTCTGCATTTTTATGATTAAAAAATAG | GTGCGCAGGATTCAAACGGAGATTAGATAGA |
|  | pHis-HbHRT | CGGAATTCAGCTTTCAAGGCACATGCATG | CGGAGCTCCACTGACTTAACCTGCT |
|  | pGADT7-HbMYB19 | CGCCATATGCAAGAACCAAAGACGAAGAG | CCTCGAGCTTTATAGACTTTGGAGGAGGGC |
|  | pGADT7-HbMYB44 | GGAATTCCATATGGTGTCGGTGAACCCCAAC | CCGCTCGAGTCAGCCTCTCACTTCTCTTGG |
|  | pGreenⅡ-HbSRPP  pGreenⅡ-HbFDP  pGreenⅡ-HbHRT  pGreenⅡ62k-HbMYB19  pGreenⅡ62k-HbMYB44 | ACTCTAGACATCCCATAACGTCTTGAGTC  ACTCTAGACTGCATTTTTATGATTAAAAAATAGTT  GCGCTCGAGTGCATTAAAATGAAAAAGTT  CGGGATCCATGCAAGAACCAAAGACGAAGAG  CGAGCTCATGGTGTCGGTGAACCCCAACCCG | TGTGTCGACAATTGAAAATTTCCTTTAAAAATCAC  TGTGTCGACGGATTCAAACGGAGATTAGATAGAA  GCGGGATCCTTTCCTTAAACCACTGACTT  CCAAGCTTGGTTATAGACTTTGGAGGAGGGCTC  CCAAGCTTGGTCAGCCTCTCACTTCTCTTGGAGC |
